# Controlling intrusive thoughts of future fears under stress

**DOI:** 10.1016/j.ynstr.2023.100582

**Published:** 2023-11-02

**Authors:** Stephanie M. Ashton, Tom Smeets, Conny W.E.M. Quaedflieg

**Affiliations:** a Department of Neuropsychology and Psychopharmacology, Maastricht University, Maastricht, the Netherlands; b Department of Medical and Clinical Psychology, Center of Research on Psychological Disorders and Somatic Diseases (CoRPS), Tilburg University, the Netherlands

**Keywords:** Intrusions, Episodic future thinking, Acute stress, Cortisol, Propranolol, Metyrapone

## Abstract

Negative outlooks of our future may foster unwanted and intrusive thoughts. To some extent, individuals have control over their ability to suppress intrusions and downregulate their frequency. Acute stress impairs intentional suppression, leading to an increased frequency of intrusions. The aim of this study was to gain insight into the mechanism underlying stress-induced impairments in intentional suppression of intrusions by investigating the combined and independent roles of the two major stress hormones, noradrenaline and cortisol. Healthy participants (*N* = 181) were administered propranolol (to block the noradrenergic response), metyrapone (to block the cortisol response), or a placebo before being exposed to the Maastricht Acute Stress Test. Intrusive thoughts of autobiographical future fears were then measured via the Imagine/No-Imagine task. Results demonstrated that the stress response was successfully altered because of the drug and stress manipulations. In all groups, repeated suppression of future fears reduced intrusions. Across the sample, an enhanced decrease over time was associated with greater attenuation of anxiety towards the related fears. The groups did not differ in the total frequency of intrusions. Though, trait anxiety increased the total number of intrusions. Our findings show that stress hormones did not influence the ability to suppress intrusions. However, our results do add support to previous research linking anxiety to memory control deficits. When using autobiographical content, future research should focus on the quality and characteristics of the individual memories to explain more of the variation observed in intentional memory control.

## 1 Introduction

Our memories define who we are and help us adapt to current and future events (Schacter et al., 2017). Unwanted thoughts, such as future worries of feared events, can intrude into awareness when we are confronted with reminders (Ashton et al., 2020; Benoit et al., 2016; Levy and Anderson, 2012). Our ability to intentionally control and downregulate the frequency of unwanted thoughts of future fears is enabled through control mechanisms similar to those engaged when we intentionally control our past memories (Benoit et al., 2016), and serves as an adaptive emotion regulation strategy to suppress and forget memories that pose a threat to our integrity and well-being (Engen and Anderson, 2018). Intentional retrieval-suppression requires conscious and instructed effort, which differs from other passive thought suppression tasks that can ironically increase the frequency of an unwanted thought, as seen in the White Bear paradigm (Wegner, 1994). The inability to control fear-related memories has been suggested to play a key role in the development and maintenance of stress-related psychopathology, such as post-traumatic stress disorder (PTSD) or mood and anxiety disorders (for meta-analysis, see Stramaccia et al., 2021). Previous research found that acute stress impairs intentional memory control (Ashton et al., 2020; Quaedflieg et al., 2020, 2022). Delineating how the stress response impairs the ability to downregulate unwanted thoughts is of critical importance to advance our theoretical understanding of stress-related symptomatology and potentially devise interventions targeting stress-related mechanisms.

Intentional control of future fears is measured through the Imagine/No-Imagine paradigm (I/NI; Benoit et al., 2016). The I/NI paradigm was adapted from the Think/No-Think paradigm (T/NT; Anderson and Green, 2001), and uses negative, autobiographical content to mimic memory control that is truer to real life. Participants first create cue-target pairs consisting of words relating to their own fears. During the next T/NT phase, participants are tasked to retrieve or suppress the target when presented with the associated cue. In this phase, involuntary retrievals can be measured on a trial-by-trial basis by asking participants to report to what extent the target comes to mind during the presentation of the cue (Levy and Anderson, 2012). A failure to prevent retrieval during no-think trials is classified as an intrusion. Over repeated suppression attempts, the frequency of intrusions decreases (Davidson et al., 2020; Gagnepain et al., 2017; Harrington et al., 2021; Hellerstedt et al., 2016; Levy and Anderson, 2012; Satish et al., 2022). The after-effects of retrieval-suppression are then tested in a final recall phase. Generally, memory performance is lower for memories that have been suppressed compared to memory for baseline items (i.e., memories not cued in the retrieval-suppression phase). The recall scores from this phase are used to determine suppression-induced forgetting (SIF; for review, see Anderson and Hulbert, 2021). SIF of neutral stimuli has been linked to reduced distress from memory intrusions following an analog trauma in the following week outside the laboratory (Streb et al., 2016). Outside of the lab, however, people are rarely motivated to retrieve memories that they have actively tried to suppress (Anderson et al., 2016), which arguably makes measuring intrusions during suppression a better metric to reflect real-life memory control compared to SIF and more closely resembles symptomatology in stress-related disorders.

Acute stress has been found to impair the intentional suppression of future fears, reflected by increased intrusions (Ashton et al., 2020). Stress alters the functioning of frontal and temporal brain areas implicated in intentional memory control (Hermans et al., 2014; Young et al., 2017). In response to a stressor, activation of the hypothalamic-pituitary-adrenal (HPA) axis results in the release of the human glucocorticoid cortisol (Joëls et al., 2011; Ulrich-Lai and Herman, 2009). In addition to the HPA axis response, activation of the rapidly acting autonomic nervous system (ANS) results in the release of noradrenaline (Ulrich-Lai and Herman, 2009). This combined acute stress response prompts the reallocation of resources to the salience network, promoting a hypervigilant state at the expense of the executive control network (Hermans et al., 2011, 2014). The dorsolateral prefrontal cortex (dlPFC) is part of the executive control network, the key neural substrate of intentional memory control (Anderson & Hanslmayr, 2014; Benoit et al., 2016), which is impaired after exposure to acute stress (McEwen and Morrison, 2013; Qin et al., 2009). We currently have some understanding of the role of acute stress-induced cortisol in suppression-induced forgetting. Previous research has demonstrated that cortisol reactivity appears to drive impairments in intentional suppression (Quaedflieg et al., 2022). Participants who elicited a cortisol response following acute stress demonstrated impaired suppression abilities, whereas stressed participants who did not elicit a cortisol response demonstrated effective suppression abilities, similar to non-stressed controls. Moreover, stress-induced cortisol increases have been associated with altered connectivity between the hippocampus and rdlPFC, which reduced the ability to suppress unwanted memories (Quaedflieg et al., 2020). This would suggest that cortisol plays a critical role in modulating intentional memory control. However, the role of noradrenaline or their combined role in intentional control of intrusions are not yet known.

This study aims to delineate the effect of stress hormones on intrusive thoughts by using pharmacological manipulations to temporarily block either of the two main stress hormone systems. First, propranolol, a non-selective β-adrenergic receptor antagonist that has been found to impair both short and long-term memory of emotionally arousing material (Cahill et al., 1994; Lonergan et al., 2013; Maheu et al., 2004), was administered to block the ANS (Black et al., 1964). This emotional memory impairment has been shown to result from blocking central as opposed to peripheral β-adrenergic receptors (van Stegeren et al., 1998). Metyrapone, which inhibits the 11- β hydroxylation reaction in the adrenal cortex, was used to block the cortisol responses induced by the HPA-axis. Metyrapone has been found to impair long-term memory for emotional arousing material (Antypa et al., 2019; Marin et al., 2011; Rimmele et al., 2015). In the current design, participants were administered either a placebo, propranolol or metyrapone, and were subsequently exposed to the Maastricht Acute Stress test. The three stress groups were compared to a no-stress placebo group. Thereafter, participants retrieved or suppressed thoughts of their own future feared events. As previous research has demonstrated that cortisol appears to drive impairments in intentional suppression (Quaedflieg et al., 2022), we hypothesised that, in comparison to propranolol and placebo, blocking the glucocorticoid response using metyrapone would block the stress-induced impairment in intentional memory control, resulting in fewer intrusions and a greater ability to downregulate intrusions over time.

## 2 Material and methods

### 2.1 Participants

The study recruited healthy participants aged between 18 and 35. The *a-priori* power calculation was performed using G*Power [α = 0.05, 1-β = 0.90] and indicated a sample of 176 participants, based on the effect size reported for the intrusion analysis in Ashton et al. (2020). A total of 217 participants were recruited and medically screened using a questionnaire. The medical screening questionnaire checked the following inclusion criteria: absence of psychiatric history (in the past 3 years); no current medical condition; no use of regular medication; smoking (<10 cigarettes per week); alcohol (<10 cups per week); BMI between 17.5 and 30 and drug use (<2 per month). Moreover, blood pressure was checked for hypotension (systolic <90 and diastolic <60), a contraindication to propranolol, during the screening and at the start of test day 1.

Thirty-two participants did not pass the medical screening and four ended their participation before the end of the procedure. All participants were compensated with course credits or money. Females were using hormonal contraceptives to control for the known influence on the cortisol stress response (Kirschbaum et al., 1999; Strahler et al., 2017). Test protocols were approved by the local medical review committee (METC_NL73481.068.20) and were conducted in accordance with the provisions of the World Medical Association Declaration of Helsinki. All participants provided informed consent.

### 2.2 Design

**Fig. 1:**
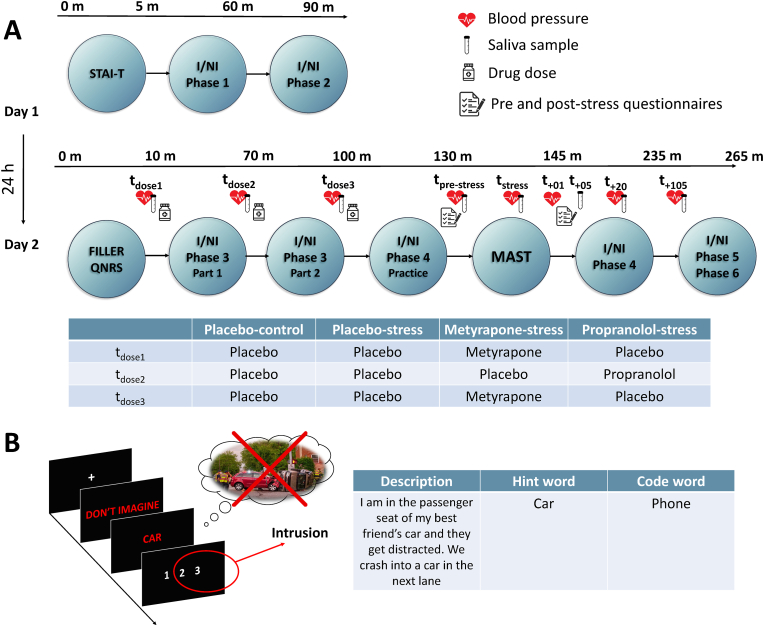
Outline of the two-day testing procedure (Panel A). Time points of drug doses (at 120, 60 and 30 min prior to the Maastricht Acute Stress Test (MAST)), blood pressure, saliva samples and questionnaires (QNRs) are indicated in between phases of the Imagine/No-Imagine task and MAST. The timeline for day 2 is denoted in minutes. Panel B presents a No-Imagine trial during phase 4 of the I/NI task for the example fear of being in a car accident. The trial begins with a fixation cross, followed by the instruction ‘Don't Imagine’, followed by a corresponding hint word, followed by the intrusion rating.

This study is a four-armed, placebo-controlled, between-subjects design. Three groups were allocated propranolol, metyrapone or a placebo, and were subsequently exposed to an acute stress induction procedure. In these groups, the drug allocation was double-blind. The fourth group was given a placebo and completed a no-stress control version of the acute stressor. Placebo was allocated to this group to ensure single blinding for the participant. This group could not be double blind as the experimenter knew the no-stress condition acted as the single control group. The test sessions were scheduled over two consecutive days (see procedure outline in Fig. 1 and methods section 2.9.). Day 1 testing took place in the morning between 08:30 and 12:00 and lasted between 1.5 and 2 h. Day 2 testing started after 11 a.m. to avoid fluctuations in the circadian rhythm of cortisol. The duration of the test day was approximately 5 h. Participants were instructed to be well rested for the test days, not to eat or drink anything but non-sparkling water 2 h before testing and to refrain from caffeine, smoking and heavy exercise on the morning of testing.

### 2.3 Drug administration

Participants received either propranolol hydrochloride (40 mg oral administration; a safe challenge that temporarily blocks β -adrenergic receptors) or metyrapone (metopirone©, 750 mg orally administered twice, a safe challenge that temporarily blocks cortisol synthesis). A starch capsule was given as the placebo. All medications were prepared by the pharmacy of UMC+ (Maastricht, the Netherlands). The tablets were not identical in appearance. To mask the medication, each dose was administered in an identical and opaque pharmacy pill bottle, so that its content was not visible to the participant or experimenter. The participant was instructed to swallow the content directly from the bottle, rather than place in their hand first.

The doses and drug timings were based on previous studies that aimed to block the acute stress response prior to the onset of a laboratory stressor (Hermans et al., 2011). Three doses were administered for all 4 groups and were timed so that the active drug groups would reach peak levels prior to exposure to the stressor (see Fig. 1 for timings). To ensure blinding, all participants took either an active drug dose or a placebo at 3 time points. Participants in the stress-placebo and no-stress placebo were given a placebo at 3 time points. Participants in the metyrapone-stress group were given 750 mg of metyrapone at doses 1 and 3, and placebo at dose 2. The bottles were labelled (dose 1, dose 2 and dose 3) by an independent researcher. To reduce possible side effects, the doses of metyrapone were given 90 min apart. Participants in the propranolol-stress group were given 40 mg of propranolol at dose 2 and placebo at doses 1 and 3. Further, all doses were administered with milk and all participants ate a yoghurt after the third dose. Feelings of nausea were reported by 2 participants in the metyrapone-stress group, although these were minor and short-lived.

### 2.4 The Maastricht Acute Stress Test

The Maastricht Acute Stress Test (MAST; Smeets et al., 2012) was used to activate the human stress response prior to phase 4 of the I/NI task (see section 2.8.). The MAST consists of a 5-min instruction phase, followed by a 10-min acute stress phase alternating between two trial types: immerse their hand in cold water (2–4 °C) and challenging mental arithmetic, whilst receiving negative feedback from the experimenter and viewing their live reaction in a video image. Participants were told that they would be videotaped throughout in order to later analyse their facial expressions during the task, although in reality these data were not recorded. The experimenter performing the MAST was independent from the experimenter present for the test day.

The no-stress control version aims to provide the same type of experience to participants but without eliciting a stress response. Participants were required to immerse their hand in lukewarm water (between 35 and 37 °C) and count continuously from 1 to 25 for mental arithmetic trials. Participants did not receive feedback and they were not video-monitored.

### 2.5 Physiological stress measures

Saliva was sampled via synthetic Salivettes (Sarstedt, Etten- Leur, the Netherlands) at 8 time points on the second test day. Four samples were measured prior to the MAST (t_dose1_, t_dose2_, t_dose3_, t_pre-stress_) one during (t_stress_) and three after (t_+05_, t_+20_, t_+105_) to obtain concentrations of cortisol and salivary alpha-amylase (sAA). A mock sample was also taken on the first test day to ensure participants had adhered to the rules set regarding eating and drinking. Samples were stored at −20 °C after collection until cortisol and sAA concentrations were determined by a commercially available chemiluminescence immunoassay with high sensitivity (IBL International, Hamburg, Germany). The intra- and inter-assay coefficients were both below 9%. Pulse, systolic and diastolic blood pressure (SBP; DBP) were recorded using an Omron 705IT (HEM-759-E; Omron Healthcare Europe BV) and measured from the right arm at 8 time points (t_dose1_, t_dose2_, t_dose3_, t_pre-stress_, t_stress_, t_+01_, t_+20_, t_+105_).

### 2.6 Subjective stress measures

Following the MAST, participants reported how stressful, painful and unpleasant they had perceived the MAST via three 100 mm Visual Analog Scales (VASs; anchors: 0 = not at all; 100 = extremely). Subjective stress was determined via the mean score.

The negative subscale of the Positive and Negative Affect Scale, short version (PANAS-SF; Watson et al., 1988) was administered at pre-stress and post-stress to determine the change in negative affect in response to the MAST. The scale has 5 items, scored on a likert scale (1 = not at all; 5 = extremely). Higher sum scores indicate increased negative affect, with possible scores ranging from 5 to 25.

### 2.7 Trait anxiety

Trait anxiety has been found to negatively influence the ability to suppress unwanted memories (Benoit et al., 2016; Marzi et al., 2014; Waldhauser et al., 2011). Therefore, trait anxiety was measured at the start of test day 2 via the State-Trait Anxiety Inventory (STAI-T; Spielberger et al., 1983). The STAI-T consists of 20 self-report items, with scores recorded on a Likert scale (1 = almost never; 4 = almost always). Higher sum scores indicate higher levels of trait anxiety, with possible scores ranging from 4 to 80.

### 2.8 Imagine/No-Imagine paradigm (I/NI)

This experiment employed an adaptation of the paradigm implemented by Ashton et al. (2020). This task comprises 6 phases: 1) Fear generation; 2) Imagination task (baseline); 3) Reminder task; 4) Retrieval-suppression task; 5) Final recall test and 6) Imagination task (post-test). Phases 1 and 2 were completed on day 1, and phases 3, 4, 5, and 6 on day 2.

#### 2.8.1 Phase 1: fear generation

Participants provided written descriptions of 18 events they feared for their future. After the medical screening, participants were given guidelines on criteria that the feared events had to meet, which they had to prepare before the first test day (see OSF for instructions given to participants). On test day 1, participants were asked to write down descriptions for each event and generate a ‘hint word’ that acted as an obvious reminder of the memory, and a ‘code word’ that acted as a personal reminder of the memory. For example, if the fear related to a car accident, the hint word could be ‘car’. This is an obvious reminder that the participant or another reader would be able to link back to the description. The code word in this instance needs to be a detail that is specific to the participant's imagination, such as what caused the car accident. If the participant imagines themselves or the driver to be distracted by a call or text, the code word could be ‘phone’. Compared to the hint word, this acts as a more subtle reminder to the participant and would not be an obvious association for someone else. Participants were instructed to write in their first language (recruitment was limited to English, Dutch or German speakers, based on language capabilities of the research team). They were asked to rate each memory on 6 likert scales measuring vividness, emotional intensity, likelihood of occurrence, distance in the future, frequency of thought and anxiety. These ratings were used to match 15 fears across the 3 stimulus types: Imagine, No-Imagine and Baseline. The remaining 3 fears that were rated lowest on the scales were used as practice items for phase 4.

#### 2.8.2 Phase 2: imagination task (baseline)

Participants were then presented with all 15 hint words in a random order. Participants were asked to vividly imagine each of the events through a first person perspective. Each trial was presented for a maximum of 60 s. Participants had the option to skip to the next trial after 40 s. At the end of each trial, participants rated their current feeling of anxiety toward the event on a 5-point Likert scale (1 = not at all; 5 = extremely).

#### 2.8.3 Phase 3: reminder task

As there were 24 h between the first and second test day, participants began day 2 with a reminder task in which they were presented with the descriptions for each of the 18 fears they had produced in phase 1. Participants were asked to recall and write down the corresponding hint and code words. They were then presented with the correct combinations. Next, participants were asked to write specific details for each of the events by describing: who they are with; when and where the event will happen; what will happen; and the senses and feelings they can imagine.

In the second part of the reminder task, participants were presented with all hint and code pairs, one by one. Participants were asked to speak aloud about each of the descriptions, describing the event in the present tense from a first-person perspective. Each trial was presented for a maximum of 60 s. Participants had the option to skip to the next trial after 40 s.

#### 2.8.4 Phase 4: retrieval-suppression task

This phase cued 10 of the 15 hint words: 5 for Imagine trials and 5 for No-Imagine trials. Five of the hint words were not cued and were used as a baseline control measure for analysis. Each trial presented an instruction cue (either ‘Imagine’ or ‘Don't Imagine’) for 1.5 s, followed by a corresponding hint word that was presented for 4 s. During ‘Imagine’ trials, participants were instructed to vividly imagine the associated fear. Moreover, their imagination should incorporate the typical detail they had produced (i.e. code word). During ‘No-Imagine’ trials, the direct suppression strategy was employed (Bergström et al., 2009). Here, participants were instructed to block out all imagination of the fear and do so without using distraction tactics, such as replacing it with another thought. If a thought did enter their mind, they were to actively push it out of awareness and keep their attention on the cue word. At the end of each trial, the question ‘How often did you imagine?’ was presented. Participants had 1.5 s to respond by pressing 1, 2 or 3 on the keyboard (1 = never; 2 = briefly; 3 = often). Participants were instructed to press 1 if the target did not come to mind at any point while the cue was on the screen. They were instructed to press 2 if the target came to mind even just momentarily, but they were able to then push it out of awareness. They were instructed to press 3 if they were unable to push the target out of mind or if the target returned one or more times after attempts at blocking the thought. Each hint was presented 14 times^1^ in a random order, with the restriction that no more than two instruction cues (either ‘Imagine’ or ‘Don't Imagine’) were shown consecutively. The task was completed in two parts of 7 repetitions, with a break in between wherein a saliva sample and blood pressure were taken.

The task began with 3 short practice rounds, each cueing one Imagine trial and two No-Imagine trials. The experimenter verbally administered diagnostic questions and discussed the participant's strategy after each round to ensure understanding and compliance with task instructions (see OSF for task instructions).

#### 2.8.5 Phase 5: final recall

Participants were presented with all 15 hint words, one by one, and responded by saying the corresponding code word aloud. The percentage of correctly recalled code words for each stimulus type was recorded (Imagine, No-Imagine and Baseline). Practice items were not cued in this task.

#### 2.8.6 Phase 6: imagination task (post-test)

As done at the end of the fear generation phase on test day 1, participants were asked to vividly imagine each of the 15 fears through a first-person perspective. Each trial was presented for a maximum of 60 s. Participants had the option to skip to the next trial after 40 s. At the end of each trial, participants rated their current feeling of anxiety toward the event on a 5-point Likert scale (1 = not at all; 5 = extremely).

### 2.9 Procedure

On test day 1, participants began by completing the STAI-T and the Stroop and digit span task (see Supplementary Results S4). Next, participants completed phases 1 and 2 of the I/NI task. On test day 2, participants began by completing filler questionnaires for approximately 10 min to accommodate to the test environment (Shields, 2020; Strahler et al., 2017). Next, the first saliva and BP measure were taken (t_dose1_), followed by the first drug dose. Participants then completed filler tasks (Stroop and digit span) followed by the first part of the reminder task (phase 3). The second saliva sample and BP were then measured (t_dose2_), followed by the second drug dose. Next, participants completed the second part of the reminder task. Saliva and BP were then measured (t_dose3_) before the third and final drug dose. Participants then began the practice phase for the I/NI before completing pre-stress questionnaires, and another saliva sample and BP (t_pre-stress_) measurement were obtained. Subsequently, participants were exposed to the stress or control version of the MAST. Saliva and BP were measured after the second trial of the MAST (t_stress_) and BP was measured again immediately after (t_+01_). Participants then completed post-stress questionnaires and a saliva sample (t_+05_), followed by phase 4 of the I/NI task, with saliva and BP measured halfway through (t_+20_). This was followed by a 1-h break and a lunch was provided. Finally, a saliva sample and BP measure (t_+105_) were taken before completing phases 5 (see Supplementary Results S3) and 6 of I/NI paradigm. Participants were debriefed at the end of the study.

### 2.10 Statistical analysis

As our primary research question was to investigate intrusions, our analysis focused on No-Imagine items. In line with previous studies (Harrington et al., 2021; Hellerstedt et al., 2016; Levy and Anderson, 2012), responses of ‘often’ accounted for a small proportion of responses (see Table 1). Therefore, responses of 2 (briefly) or 3 (often) during No-Imagine trials were classified as an intrusion (Ashton et al., 2020; Ashton et al., 2023; Benoit et al., 2015; Castiglione et al., 2019; Castiglione and Aron, 2020; Chen et al., 2022; Gagnepain et al., 2017; Legrand et al., 2020; Levy and Anderson, 2012; Nishiyama and Saito, 2022; Satish et al., 2022; van Schie and Anderson, 2017). We quantified two measures of intrusions: the total frequency (%) and the Index of Intrusion control (IIC; Ashton et al., 2023) to measure the time course of intrusion frequencies over repeated suppression attempts. Increasingly negative values indicate a larger decrease in intrusions over time. Due to the high number of repetitions and break halfway through the task, we calculated both the total frequency and IIC measure across all blocks and separately for part 1 and part 2.

**Table 1:** Intrusion responses from the retrieval-suppression phase for No-Think trials. The proportion (%) of valid trials (i.e., after removing trials with no response) are reported for each response type (Never, Briefly, Often) for each group.

Condition	Never	Briefly	Often
Placebo-control	74.62	23.00	2.38
Placebo-stress	76.61	21.12	2.27
Metyrapone-stress	76.15	22.05	1.80
Propranolol-stress	71.02	25.46	3.52

Anxiety indexes were calculated from the imagination task (phase 2 and 6), for Imagine, No-Imagine and Baseline items ((pre score – post score)/pre-score). Higher positive scores indicate a greater decrease in anxiety.

The data were checked for normality. A log-transformation was performed to account for skewed cortisol and sAA values. In cases of violated normality or sphericity, adjusted Welch's *F* ratios and Greenhouse-Geisser corrected values are reported, respectively. Follow-up tests applied Tukey corrections for multiple comparisons, or Games-Howell in case of unequal variances. The behavioral data were checked for outliers using the IQR*3 method. Two-tailed *p*-values are reported. Null findings are supplemented with corresponding Bayesian analyses (BF^01^).

From the sample that completed the full procedure (*N* = 181), three participants were excluded due to measurement error (i.e., the wrong stimuli were cued during phase 4). Participants with ≥2 missing intrusion trials in any 1 of the 14 blocks during phase 4 were excluded from the analysis (*n* = 15). Overall, missing trials across the sample were low (1.8%). The final sample included for the analyses is *N* = 164 (placebo-control: *n* = 41 (male = 20); placebo-stress: *n* = 42 (male = 17); propranolol-stress: *n* = 40 (male = 16); metyrapone-stress: *n* = 41 (male = 16). The distribution of sex across the groups did not differ (*X*^2^_(1,3)_ = 1.03, *p* = .80). Furthermore, age (*M* = 21.86; *SD* = 3.06) and BMI (*M* = 22.13; *SD* = 2.30) did not differ between the groups (both *p*'s > 0.38).

## 3 Results

### 3.1 Successfully altered stress responses after drug manipulations

**Fig. 2:**
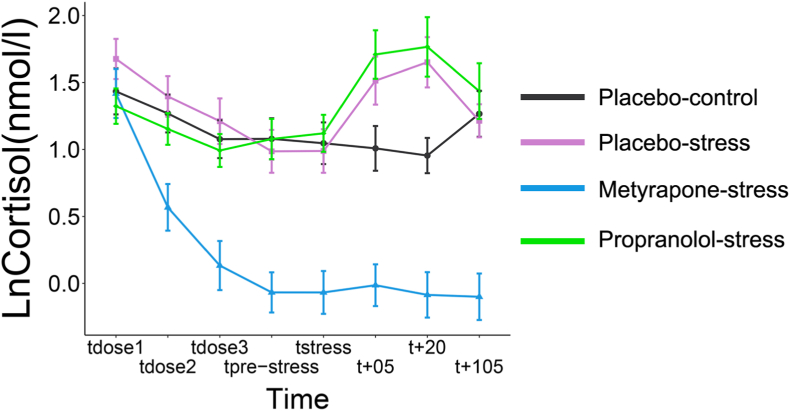
Log-transformed values for cortisol in response to the drug and stress manipulations for the 4 groups across 8 time points. The graphs display means and 95% confidence intervals.

Cortisol values differed significantly throughout test day 2 between the groups (Time*Group: *F*_(10.94, 565.25)_ = 13.35, *p* < .001, ηp^2^ = 0.21; see Fig. 2). Follow-up tests were performed at each time point. Participants in the metyrapone-stress group had lower cortisol compared to all groups at t_pre-stress,_ t_stress_, t_+05_ and t_+20_ (all *p's* < 0.001). Therefore, metyrapone produced the desired effect on the stress response by suppressing cortisol throughout the I/NI task.

At t_+05_ and t_+20_, placebo-control participants had decreased cortisol compared to participants in the propranolol-stress and placebo-stress group (both *p*'s < 0.001), whereas participants in the placebo-stress and propranolol-stress group had comparable cortisol levels (both *p*'s > 0.36; BF^01^'s > 1.55). No other group differences were observed t_prestress_ and t_stress_ (all *p*'s > .16; BF^01^'s > 0.66; see Supplementary results S1 for follow ups at other time-points). Thus, the MAST successfully increased cortisol in the placebo-stress and propranolol-stress groups in comparison to placebo-control.

**Fig. 3:**
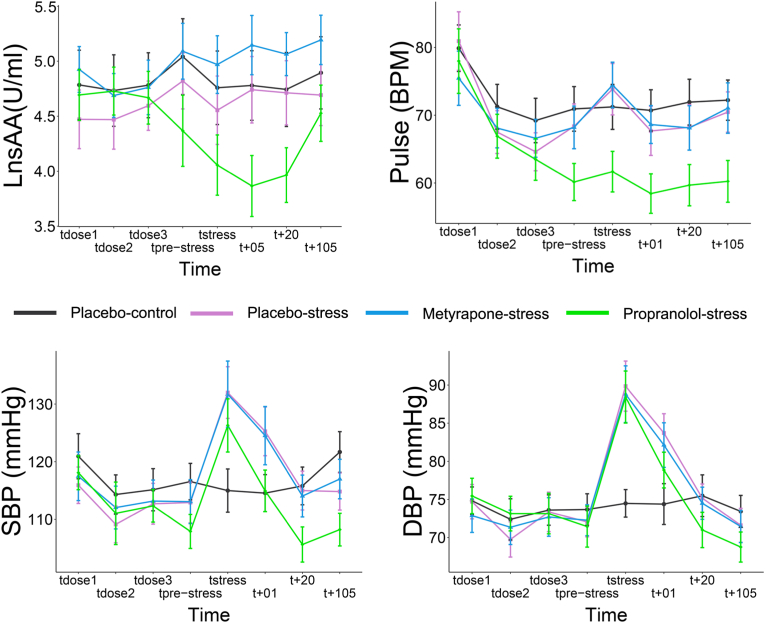
Noradrenergic responses to the drug and stress manipulations. The graphs display means and 95% confidence intervals of salivary alpha amylase (sAA), pulse (beats per minute), systolic (SBP) and diastolic (DBP) blood pressure for the 4 groups across 8 time points. The values for sAA are log-transformed.

Regarding the noradrenergic response,^2^ sAA differed significantly throughout test day 2 between the groups (Time*Group: *F*_(2.33, 895.41)_ = 8.21, *p* < .001, ηp^2^ = .14; see Fig. 3). At t_pre-stress_ and t_stress_, the propranolol-stress group had decreased sAA compared to placebo-controls and metyrapone-stress (all *p*'s < .008), but comparable levels to placebo-stress (both *p's* > 0.079), BF^01^'s > 0.35). There were no differences between placebo-control, metyrapone-stress and placebo-stress at these time points (all *p*'s > 0.20, BF^01^'s > 0.71). At t_+05_ and t_+20_, propranolol-stress had decreased sAA compared to all groups (all *p*'s < 0.001), whereas all other groups had comparable levels (all *p*'s > 0.20, BF^01^'s > 0.75). Therefore, propranolol produced the desired effect on the stress response by suppressing noradrenergic activity. However, the MAST did not successfully increase sAA for placebo-stress or metyrapone-stress in comparison to placebo-control.

In addition, both systolic (SBP) and diastolic (DBP) blood pressure^3^ differed throughout day 2 across the groups (Time*Condition; SBP: *F*_(13.39, 771.48)_ = 16.49, *p* < .001, ηp^2^ = .24; DBP: *F*_(14.58, 753.11)_ = 14.67, *p* < .001, ηp^2^ = .22; see Fig. 3). Follow up results for SBP showed that at t_prestress_, the propranolol-stress group had lower values than placebo-controls (*p* = .002), whereas all other groups were comparable (all *p*'s > 0.13, BF^01^'s > 0.54). At t_stress_, SBP values of the placebo-control group were lower than all other groups (all *p*'s < .004), whereas all other groups were comparable (all *p*'s > 0.31, BF^01^'s > 1.09). At t_+01_, the values of placebo-control and propranolol-stress were both lower than placebo-stress and metyrapone-stress (all *p*'s < 0.014). Placebo-controls versus propranolol-stress and metyrapone-stress versus placebo-stress were comparable (both *p*'s > 0.99, BF^01^'s > 4.23). Therefore, the MAST successfully increased blood pressure in the stress groups compared to controls and propranolol lowered blood pressure prior to the retrieval-suppression task. See Supplementary Results S2 for follow-up tests at other time points for SBP and DBP.

Pulse differed throughout test day 2 across the groups (Time*Condition; *F*_(13.75, 723.88)_ = 91.80, *p* < .001, ηp^2^ = .37; see Fig. 3). Follow up results showed that there were no group differences at t_dose1_ and t_dose2_ (all *p*'s < 0.21, BF^01^'s > 5.04). Participants in the propranolol-stress group had a lower heart rate than controls at t_dose3_ (*p* = .034). All other groups were comparable (all *p*'s > 0.13, BF^01^'s > 0.58). Participants in the propranolol-stress group had a lower heart rate than all groups at each subsequent time point (all *p*'s < .002). There were no differences between the other 3 groups at any time point (all *p*'s > 0.13, BF^01^'s > 1.36).

### 3.2 Blocking cortisol or noradrenaline does not change the subjective experience of acute stress

Participants differed in their subjective experience^4^ of the MAST (*F*_(3, 84.38)_ = 187.79, *p* < .001, ηp^2^ = .68; see Table 2). Participants in the placebo-control group rated the MAST as subjectively less stressful than those in the 3 stress groups (all *p*'s < 0.001), who did not differ in their ratings (all *p*'s > 0.83; BF^01^'s > 3.18).

**Table 2:** Trait anxiety, subjective stress and negative affect in response to the MAST for 4 groups. Means and standard deviations are presented.

Condition	STAI-T		VAS		PANAS			
					Pre-stress		Post-stress	
	*M*	*SD*	*M*	*SD*	*M*	*SD*	*M*	*SD*
Placebo-control	36.39	6.03	9.22	10.82	5.78	1.13	5.85	1.51
Placebo-stress	36.67	7.97	64.64	19.07	6.14	1.56	9.20	3.69
Metyrapone-stress	36.68	9.14	65.85	20.02	5.90	1.77	8.48	3.52
Propranolol-stress	36.42	7.58	68.03	16.93	6.03	1.29	8.41	2.93

**Note:** the ranges for each scale are: STAI-T: 20–80; VAS: 0–100; PANAS: 5–25.

Negative affect increased for participants in the 3 stress groups compared to placebo-controls (Time*Condition: *F*_(3, 158)_ = 10.88, *p* < .001, ηp^2^ = .17). Follow-up tests revealed no significant differences between the groups prior to the MAST (*F*_(3, 158)_ = .48, *p* = .70, ηp^2^ = .009, BF^01^ = 17.71). At the post-stress measure, the groups differed significantly (*F*_(3, 80.79)_ = 17.48, *p* < .001 ηp^2^ = .15). Participants in the 3 stress groups had increased negative affect compared to placebo-controls (all *p*'s < 0.001) and this did not differ between the 3 stress groups (all *p*'s > 0.85; BF^01^'s > 2.91).

### 3.3 Intentional suppression of future fears reduces intrusions over time

**Fig. 4:**
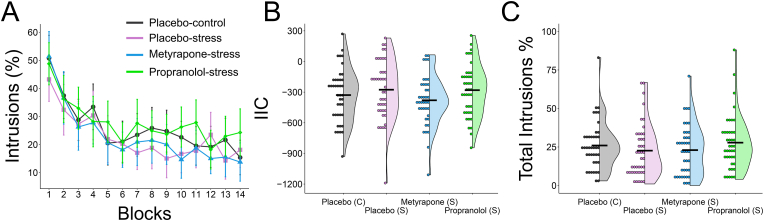
The frequency of intrusions across repeated suppression attempts (Panel A). The 4 groups decrease equally between blocks 1–7, whereas no change over time was observed between blocks 8–14. Panel B and panel C display the means for each group (placebo-control, placebo-stress, metyrapone-stress, and propranolol-stress) for the IIC and total intrusions, respectively. The split violin plots display the distribution of the data, group means (indicated by the black bars) and individual data points.

A repeated-measures ANOVA was performed to investigate the time course of intrusions across the 14 blocks of the retrieval-suppression task. Over repeated suppression attempts, the frequency of intrusions reduced (Time: *F*_(10.60, 1696.32)_ = 38.41, *p* < .001, ηp^2^ = .19). This effect was similar across the groups (Time*Group: *F*_(31.81, 1696.32)_ = 1.13, *p* = .28, ηp^2^ = .021, BF^01^ > 100; see Fig. 4). In line with this finding, the IIC and total intrusion frequency did not differ between the groups (IIC: *F*_(3, 160)_ = 1.40, *p* = .25, ηp^2^ = .025, BF^01^ = 6.33; Intrusion frequency: *F*_(3, 160)_ = 0.89, *p* = .45, ηp^2^ = .016, BF^01^ = 10.97). As per our previous work (Ashton et al., 2020), we explored whether participants in the placebo-stress group reported more intrusions than placebo-controls. Both groups reported a similar frequency (*t*_(81)_ = 0.92, *p* = .36, *d* = .22, BF^01^ = 3.02).

We next looked into part 1 and part 2 of the task separately. Whereas intrusions decreased over repeated trials in part 1 (*F*_(6, 960)_ = 51.023, *p* < .001, ηp^2^ = .24), this downregulating effect plateaued during part 2 (*F*_(5.55, 888.75)_ = 1.67, *p* = .13, ηp^2^ = .010, BF^01^ = 98.06). This effect was similar across the groups (Time*Group: both *p*'s > 0.11, BF^01^'s > 39.70). In line with these findings, the total frequency of intrusions and IIC did not differ for part 1 or part 2 across the groups (all *p*'s > 0.18, BF^01^'s > 4.44).

As no group differences were observed for intrusions, we explored whether trait anxiety may act as an individual factor that causes variation in intentional suppression. No difference was observed in trait anxiety (STAI-T) between the groups (*F*_(3,87.83)_ = 0.086, *p* = .97, ηp^2^ = .002, BF^01^ = 28.73). As such, we performed two linear regression analyses across the full sample to predict the total number of intrusions and IIC. Trait anxiety was found to marginally increase the frequency of intrusions (*F*_(1,162)_ = 3.90, *p* = .050, R^2^ = .024) but not the IIC (*F*_(1,162)_ = 2.58, *p* = .11, R^2^ = .016, BF^01^ = 1.81).

### 3.4 Intrusions are associated with increased anxiety for suppressed fears

Based on the effects observed in the original use of the paradigm (Benoit et al., 2016), we next tested whether anxiety for future fears differed between stimulus types.^5^ The repeated measures ANOVA included the anxiety indexes of the stimulus types Imagine, No-Imagine and Baseline as the within-subjects factor. Results revealed that the change in anxiety scores did not differ between stimulus types (*F*_(2, 312_) = 2.36, *p* = .096, ηp2 = .015; BF^01^ = 4.71) and this effect did not differ across the groups (*F*_(6, 312)_ = 0.76, *p* = .60, ηp2 = .014, BF^01^ = 50.41). Trait anxiety was not found to influence the anxiety indexes for Imagine, No-Imagine or Baseline fears (all *p*'s > 0.28 all BF^01^'s > 3.43).

We then explored whether intrusion measures were associated with subsequent anxiety scores for suppressed items. As no group differences were observed and no change in IIC was found in part 2, correlations were performed across the total sample for part 1 only. The IIC_part1_ was weakly correlated with the anxiety index (*r*_(160)_ = -.17, *p* = .036), demonstrating that an increased ability to downregulate intrusions may be associated with increased attenuation of anxiety. There was no association between the total frequency of intrusions and anxiety (*r*_(160)_ = .006, *p* = .94, BF^01^ = 10.08).

## 4 Discussion

This study investigated the combined and independent roles of the two main stress hormones in the ability to intentionally control intrusive thoughts under stress. Participants completed the Imagine/No-Imagine task. First, they generated descriptions of future feared events. The following day, participants were given propranolol, metyrapone or a placebo and were then exposed to the stress or control version of the MAST. During the retrieval-suppression task, participants downregulated the frequency of intrusions over repeated suppression attempts. This effect was comparable for each group. Furthermore, the groups did not differ in the total frequency of intrusions reported. However, higher trait anxiety was found to increase the total frequency of intrusions. In addition, enhanced intrusion control during the first half of the retrieval-suppression task was associated with greater attenuation of anxiety for suppressed fears.

Metyrapone and propranolol blocked the stress responses by producing lower concentrations of cortisol and sAA, respectively, following the MAST. The MAST successfully induced acute stress (Smeets et al., 2012; Quaedflieg et al., 2017; Shilton et al., 2017) by increasing cortisol in the placebo-stress and propranolol-stress groups compared to the placebo-control condition. Participants in the placebo-control, metyrapone-stress and placebo-stress groups produced comparable sAA levels during and after the MAST. This contrasts previous studies, in which the MAST has been found to increase sAA (Smeets et al., 2012; Quaedflieg et al., 2017). This discrepancy might be due to the food consumed in the current study. To reduce possible side effects of metyrapone, the doses were taken with milk and participants ate yoghurt after the third dose, which are known to increase sAA (Simões et al., 2021). However, the blood pressure measures clearly demonstrate an effect of stress on the noradrenergic response. Future research could take into account continuous measures of heart rate variability or skin conductance, which could provide deeper insight into the involvement of individual adrenergic stress responses. Moreover, alternative pharmacological approaches, such as enhancing central noradrenergic activity through yohimbine (O'Carroll et al., 1999), could help to establish a true null result relating to the role of stress hormones. In addition, α1 receptor antagonists (such as Prazosin) have been linked to improving inhibitory functions of the PFC (Ramos and Arnsten, 2007), which could have implications for improving intrusion control.

It should be noted that we only included women using hormonal contraceptives, which can produce differences in cortisol reactivity after an acute stressor (Gervasio et al., 2022). A future study would need to create a balanced design in order to compare females using hormonal contraceptives versus females in the luteal phase of their cycle. Furthermore, including an equal number of males and females in each group would allow for investigation of sex differences in the stress response.

Subjective stress and negative affect were increased for all 3 stress groups, regardless of the drug manipulation. This is in line with previous research showing similar subjective responses to stress after allocation of metyrapone, propranolol or placebo (Hermans et al., 2011). Comparable groups on the subjective level allowed us to more reliably attribute any behavioral differences to changes in the biological acute stress response. However, observing changes in subjective stress would provide insight into the impact of conscious stress on the ability to suppress intrusions. As intrusions are a conscious phenomenon, methods aimed at stress reduction, such as breath awareness (Hirshberg et al., 2018), may have a positive effect on intentional memory suppression after exposure to acute stress.

In support of previous findings, the frequency of intrusions decreased after repeated suppression attempts (Gagnepain et al., 2017; Hellerstedt et al., 2016; Levy and Anderson, 2012). This effect was observed for the first half of the task (blocks 1–7), but not for the second half (blocks 8–14). This would suggest that the ability to suppress intrusions remains stable once a certain threshold is reached. However, no difference was observed between the groups. Unlike in previous findings (Ashton et al., 2020), stressed participants did not report more intrusions overall than placebo-control participants. Compared to control participants in the previous study, controls in the current study reported approximately 15% more intrusions overall, resulting in no difference between groups. The higher intrusion rate in the control condition could be because participants were exposed to the fears more often and had more tasks relating to building a vivid and clear picture of the event. This strengthened the consolidation of the events further and subsequently could have made it more difficult for participants to suppress. This is in line with previous work showing that consolidated memories become more resistant to intentional suppression (Liu et al., 2016).

The current findings that revealed no group differences in intrusion control are also in accordance with findings in patients with PTSD that have demonstrated the same intrusion control ability and frequency of intrusions as control participants (Mary et al., 2020). On the neural level, control participants had decreased functional coupling between the dlPFC and hippocampus during suppression. It could be the case that it is not the number of intrusions that differentiates between adaptive and maladaptive coping, but rather by how they are experienced (Kleim et al., 2013). Future research should further investigate what aspects of fears or memories influence the ability to suppress them. In order to account for the difference in emotional arousal or vividness of feared events within participants, the ratings provided during the fear generation task were used to match items across the different stimulus types to create an even distribution. This approach, although fitting for the current design, still does not take into account the variability in salience of fears between participants. Previous memory control research has found that negatively valenced images are reported as more intrusive when compared to neutral (Davidson et al., 2020). Moreover, images that evoke sadness have been found to be more intrusive when compared to images that evoke disgust (Legrand et al., 2020). Looking at intrusions outside of the lab, the type of emotion linked to intrusions has been able to differentiate intrusions experienced by individuals with PTSD versus without. Intrusions in PTSD were associated with increased feelings of fear, helplessness, anger and shame (Kleim et al., 2013). In order to explain more of the observed variance, future paradigms could be adapted to investigate which aspects of negative memories make them difficult to suppress by analysing fears on an item level and focusing on intrusion quality instead of quantity.

Attenuation of anxiety for suppressed fears was associated with an increased ability to downregulate intrusions. In line with previous findings (Benoit et al., 2016), increased trait anxiety was found to influence intentional suppression through increasing intrusion frequency. Future research should investigate which mechanisms of anxiety may contribute to this variation. Fearful thoughts in anxiety may develop from aversive associative learning, whereby individuals form maladaptive predictive relationships between events or other stimuli with aversive outcomes (Franke et al., 2021; Krypotos et al., 2015). This difference in associative learning may make memories more resistant to suppression (Chalkia et al., 2023). Moreover, individuals with heightened anxiety may benefit more from pharmacological interventions compared to those with lower anxiety. Propranolol is a common treatment method for anxiety disorders (Steenen et al., 2016) and has been shown to decrease fear in PTSD (Brunet et al., 2018). Propranolol may be more effective in different contexts (i.e., in the absence of stress) within a clinical sample. Future research could investigate whether propranolol is able to help reduce intrusions in individuals with heightened anxiety.

Increased trait anxiety has also been linked to a reduced ability to attenuate anxiety for suppressed fears (Benoit et al., 2016), though this is not supported by the current results. The difference in results could be due to methodological differences. The anxiety index in the current study compares the difference between pre- and post-scores of the suppressed fears from the No-Imagine condition. In the original use of the paradigm (Benoit et al., 2016), only the anxiety post-scores were used and compared the difference between anxiety of suppressed (No-Imagine) fears to fears in the Baseline condition. Our approach better accommodates the uniqueness of the fears (i.e., by investigating the change in anxiety for the same fears, rather than comparing anxiety ratings of different fears).

Previous studies have investigated the role of individual differences in memory control abilities. An enhanced ability to suppress intrusions is associated with higher trait mindfulness (Ashton et al., 2023) and cognitive control (Chen et al., 2022), whereas ruminative tendencies (Fawcett et al., 2015), dissociative traits (Wessel et al., 2005) and neuroticism (Ryckman and Lambert, 2015) have been found to impair memory control (as reflected by a reduced SIF effect). Other environmental factors may also play a role, as sleep deprivation has resulted in an impairing effect on suppressing intrusions (Harrington et al., 2021). More studies are needed that measure baseline psychological characteristics in both healthy and clinical samples before we are to gain a deeper insight into individual factors that cause variability in intentional suppression of intrusions.

Using autobiographical content in retrieval-suppression tasks helps build a bridge between intentional suppression observed in the laboratory and memory control as experienced in real-life. Our results do not add support for the association between stress-induced impairments in intentional suppression, but do add support for the association between trait anxiety and impaired memory control (Marzi et al., 2014). When using autobiographical content, future research should focus on the quality and characteristics of the individual memories. Considering how these thoughts are experienced outside of the lab may affect their ability to suppress them in the lab.

## CRediT authorship contribution statement

**Stephanie M. Ashton:** Conceptualization, Investigation, Formal analysis, Visualization, Writing – original draft. **Tom Smeets:** Conceptualization, Writing – review & editing. **Conny W.E.M. Quaedflieg:** Funding acquisition, Conceptualization, Supervision, Resources, Investigation, Writing – review & editing.

## Declaration of competing interest

The authors declare that they have no known competing financial interests or personal relationships that could have appeared to influence the work reported in this paper.

## Data Availability

The data is accessible via OSF.

## Footnotes

1 EEG recordings were measured during this task, therefore the number of repetitions is more than observed in a typical retrieval-suppression task in order to produce sufficient power for EEG analysis (see Satish et al., 2022). The EEG results are not presented in this paper.

2 sAA could not be determined for a total of 5 samples across 3 participants.

3 Blood pressure and pulse were not recorded for one participant due to a technical fault with the device.

4 Two participants had missing data at the post-stress questionnaire and are removed from this part of the analysis.

5 Four additional participants were excluded from this analysis due to missing responses on the pre-anxiety (*n* = 3) or post-anxiety (*n* = 1).
