# Supplementary material for: Controlling intrusive thoughts of future fears under stress
Source: Neurobiol Stress. 2023 Nov 2;27:100582. doi: 10.1016/j.ynstr.2023.100582 (PMC10656271; doi:10.1016/j.ynstr.2023.100582)
Supplement: Multimedia component 1 [file mmc1.docx]

**Supplementary results**

*S1: Follow up tests for Cortisol and sAA at t_dose1_, t_dose2_, t_dose3_ and t_+105_*

Follow up tests for cortisol showed that, at t_dose1_ and t_dose2_, participants in the placebo-stress group had increased cortisol levels compared to participants in the propranolol-stress (both *p’s* > .024). No significant differences were observed between other groups at t_dose1_ and t_dose2_ (all *p*’s > .13; BF^01^’s > 0.60). At t_dose3_ and t_+105_, participants in the placebo-control, placebo-stress and propranolol-stress groups had comparable cortisol levels (all *p*’s > .16, BF^01^’S > 0.66).

Follow up tests for sAA revealed that there were no significant differences between the groups at t_dose1_, t_dose2_ and t_dose3_ (all *p*’s > .090, BF^01^’s > 2.3). At t_+105_, metyrapone-stress had increased sAA compared to placebo-stress and propranolol-stress (both *p*’s < .048). All other groups were comparable (all *p*’s > .23, BF^01^’s > 1.10).

*S2: Systolic and diastolic blood pressure increased in response to the MAST*

Follow-up tests at each time point revealed there was no difference between the groups in SBP or DBP at t_dose1_ t_dose2_ or t_dose3_ (all *p*’s > .21, BF^01^’s > 5.03). For SBP, at t_+20_, propranolol-stress was lower than all groups (all *p*’s <.002). All other groups were comparable (all *p*’s > .88, BF^01^’s > 3.44). At t_+105_, propranolol-stress was lower than all groups (all *p*’s < .021). Placebo-stress was lower than placebo-controls (*p* = .016), whereas other groups were comparable (both *p*’s > .18, BF^01^’s > 0.88).

For DBP, at t_prestress_, there were no differences between groups (*p* = .52, BF^01^ = 12.78). At t_stress_, placebo-controls were lower than all groups (all *p*’s < .001), whereas all other groups were comparable (all *p*’s > .92, BF^01^’s > 3.68). At t_+01_, placebo-controls were lower than placebo-stress and metyrapone-stress (both *p*’s < .001). Propranolol-stress was lower than placebo-stress (*p* = .043). All other groups were comparable (all *p*’s > .074, BF^01^’s > 0.28). At t_+20_, propranolol-stress was lower than placebo-controls and placebo-stress (both *p’s* < .047), whereas all other groups were comparable (all *p*’s > .13, BF^01^’s > 0.49). At t_+105_, propranolol-stress had lower DBP than controls (*p* = .009), whereas all other groups were comparable (all p’s > .21, BF^01^’s > .86). Therefore, the MAST had a similar effect on diastolic blood pressure for all 3 stress groups.

*S3: Consolidated memories are resistant to suppression-induced forgetting and unaffected by stress*

It is well-established that suppression of newly acquired memories can lead to subsequent forgetting (Anderson & Hulbert, 2021). However, investigating consolidated memories may act as a more applicable measure to real-life memory control. Consolidated and aversive memories have been found to become more resistant to suppression, with the SIF effect absent when tested the day after encoding (Liu et al., 2016). However, this effect has not been tested on autobiographical content to see if this applies to real-life, salient memories. Furthermore, how stress affects this process is not known.

The SIF index was calculated by subtracting the recall of No-Imagine items from Baseline items and then dividing by Baseline items ((Baseline – No-Imagine) / Baseline; see Ashton et al., 2020; Hellerstedt et al., 2016; Quaedflieg et al., 2022). This results in a subject-specific measure of forgetting relative to baseline memory performance, with higher positive index scores indicating increased forgetting.

As a manipulation check for the I/NI task, the SIF index was assessed in the control condition using a one-sample *t*-test. Results showed that the SIF index did not differ significantly from the test value 0 (*t*_(40)_ = -0.75 *p* = .46, *d* = 0.18; BF^01^ = 6.23). This is in line with previous findings that do not observe a SIF effect for consolidated memories (Liu et al., 2016). Furthermore, no significant difference was found between the groups (*F*_(3,160)_ = 1.23, *p* = .30, ηp^2^= 0.023, BF^01^ = 7.31; see Fig. S1).


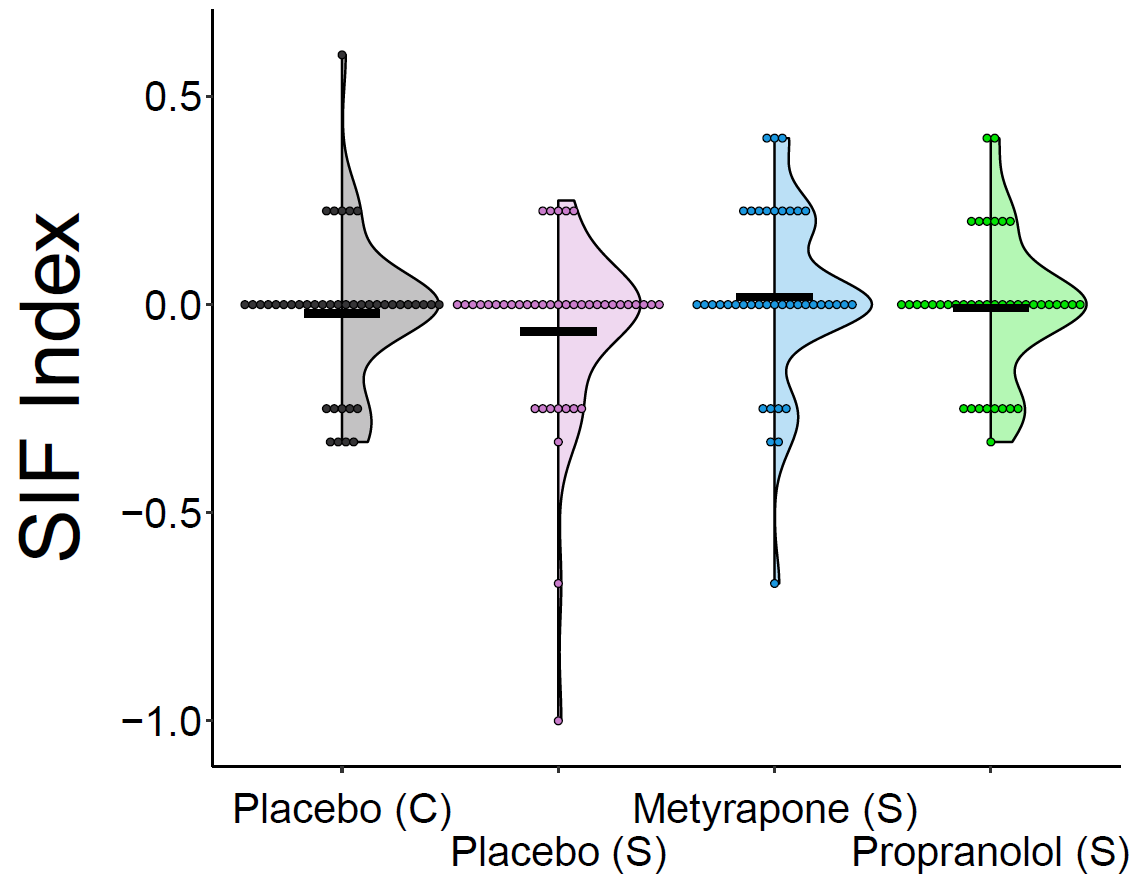


**Figure S1:** Suppression-induced forgetting (SIF) scores for the 4 groups: placebo-control; placebo-stress; metyrapone-stress and propranolol-stress. Higher index scores indicate increased forgetting. The split violin plots display the distribution of the data, group means (indicated by the black bars) and individual data points.

Previous studies using the I/NI paradigm (Ashton et al., 2020; Benoit et al., 2016) observed that suppression of future feared events lead to subsequent forgetting. In these studies, the cue-target pairs were generated and encoded on the same day as retrieval-suppression. For the current study, encoding was performed the day prior to the stressor and I/NI task to optimize the design for measuring intrusion control. In addition, participants recalled all cue-target pairs using a reminder task on day 2, making the memories stronger. Furthermore, the current study used fewer items (5 per stimulus type). This aimed to reduce variability in the strength of fears produced by participants. However, this produces smaller margins for observing differences between the forgetting of suppressed and baseline items (Satish et al., 2022). Moreover, the current study performed the final recall test 1 hour after the retrieval-suppression phase. Past research has shown that a delayed recall of 3.5 hours diminished the SIF effect (Davidson et al., 2020). The results of the current study could show that the SIF effect may diminish at an even faster rate, or could be attributed to the consolidation process that makes them stronger and more difficult to suppress.

*S4: Executive Functioning did not moderate the effect of stress on intentional suppression*

Individual differences in the ability to regulate intrusive memories may be influenced by pre-existing differences in executive control (Ashton et al., 2023; Levy & Anderson, 2012). The Stroop and digit span task were administered to measure inhibition and working memory, respectively. Low working memory capacity has been found to moderate the impairing effect of acute stress on suppression-induced forgetting (Ashton et al., 2020). As such, we aimed to replicate this effect by comparing the placebo-control and placebo-stress groups only. In addition, we investigated whether baseline inhibition moderates the effect.

Both tasks were administered via Inquisit 6 (2021). The Stroop task (Stroop, 1935) cued names of colours, written in coloured text. Participants responded by naming the colour of the word rather than the meaning of the word. The task cued 3 trial types: congruent, incongruent and control. The words were written in either the same colour as the word (congruent trials) or in a different colour than the word (incongruent trials). Control trials presented coloured blocks. Participants responded by indicating the colour of the word or block by pushing the corresponding button. We calculated an interference score (van der Elst, 2006), based on the reaction time of correct responses for incongruent, congruent and control trials (Incongruent – ((Control+Congruent) /2)). The scores were then multiplied by -1, so that higher scores are indicative of decreased interference, reflecting reduced increased inhibitory control.

The backward version of the digit span task (Woods et al., 2011) audibly cued sequences of digits, and participants had to recall them in the reverse order by selecting the correct sequence via mouse click. The length of the sequence would increase or decrease by 1 digit, depending on whether or not the participant recalled the sequence correctly. The task began with 2 practice trials with feedback. The main task was assessed over 14 trials, starting at a 2-digit sequence. The final digit span score was determined from the last length the participants could recall correctly before making two consecutive errors.

### Two moderation analyses were used to test the direct effect of condition (placebo-control vs. placebo-stress) on SIF and its moderation by executive functioning (digit span and Stroop).

The overall models did not prove to be significant (Digit span: *F*_(3,85)_ = 2.09, *p* = .11, R² = 0.07, BF^01^ = 2.57; Stroop: *F*_(3,85)_ = 0.57, *p* = .64, R² = 0.02, BF^01^ = 1.60). Therefore, we did not replicate the moderating effect of working memory found in our previous study (Ashton et al., 2020).

We next explored whether working memory or inhibition would moderate the effect of stress on intrusion control (IIC and total frequency) or the anxiety index for suppressed items. None of the models were significant (all *p*’s > .18, BF^01^’s > 2.38).

The relationship between executive functioning and intentional memory control has been mixed. Individuals with low working memory capacity have been found to be more susceptible to the detrimental effects of acute stress on SIF (Ashton et al., 2020) and other forms of higher cognitive functioning, such as goal-directed behaviour (Otto et al., 2013; Quaedflieg et al., 2019). In the absence of stress, other studies have found no association between working memory and memory control (Ashton et al., 2023; Waldhauser et al., 2015). Inhibition has been argued to be the mechanism behind intentional suppression (Levy & Anderson, 2008), and recent findings have observed that increased inhibition has been associated with an increased ability to control memory intrusions (Ashton et al., 2023). Despite this, no link has been found in the current study which does not allow us to draw conclusions about the role of inhibition and the effect of acute stress.

**References**

Anderson, M.C., Hulbert, J.C., 2021. Active Forgetting: Adaptation of Memory by Prefrontal Control. Annu Rev Psychol. 72, 1–36.

Ashton, S.M., Benoit, R.G., Quaedflieg, C.W.E.M., 2020. The impairing effect of acute stress on suppression-induced forgetting of future fears and its moderation by working memory capacity. PNEC. 120, 104790.

Ashton, S.M., Sambeth, A., Quaedflieg, C.W.E.M., 2023. A mindful approach to controlling intrusive thoughts. Sci Reports. 13, 10966.

Benoit, R.G., Davies, D.J., Anderson, M.C., 2016. Reducing future fears by suppressing the brain mechanisms underlying episodic simulation. PNAS. 113(52), E8492-E8501.

Davidson, P., Hellerstedt, R., Jönsson, P., Johansson, M., 2020. Suppression-induced forgetting diminishes following a delay of either sleep or wake. J Cogn Psychol. 32(1), 4–26.

Hellerstedt, R., Johansson, M., Anderson, M.C., 2016. Tracking the intrusion of unwanted memories into awareness with event-related potentials. Neuropsychologia 89, 510-523.

Levy, B.J., Anderson, M.C., 2008. Individual differences in the suppression of unwanted memories: The executive deficit hypothesis. Acta Psychologica 127, 623–635.

Liu, Y., Lin, W., Liu, C., Luo, Y., Wu, J., Bayley, P.J. et al., 2016. Memory consolidation reconfigures neural pathways involved in the suppression of emotional memories. Nat Commun. 7, 13375.

Otto, A.R., Raio, C.M., Chiang, A., Phelps, E.A., Daw, N.D., 2013. Working-memory capacity protects model-based learning from stress. PNAS. 110(52), 20941-20946.

Quaedflieg, C.W.E.M., Stoffregen, H., Ashton, S.M., 2022. Cortisol reactivity impairs suppression-induced forgetting. PNEC. 142, 105774.

Quaedflieg, C.W.E.M., Stoffregen, H., Sebalo, I., Smeets, T., 2019. Stress-induced impairment in goal-directed instrumental behaviour is moderated by baseline working memory. Neurobiol Learn Mem. 158, 42-49.

Satish, A., Hellerstedt, R., Anderson, M.C., Bergström, Z.M., 2022. EEG evidence that morally relevant autobiographical memories can be suppressed. Cogn Affect Behav Neurosci. 22, 1290-1310.

Stroop, J.R., 1935. Studies of interference in serial verbal reactions. J Exp Psychol. 18, 643-662.

Van der Elst, W., Van Boxtel, M.P.J., Van Breukelen, G.J.P., Jolles, J., 2006. The Stroop Color-Word Test: Influence of Age, Sex, and Education; and Normative Data for a Large Sample Across the Adult Age Range. Assessment 13, 62-79.

Waldhauser, G.T., Bäuml, K.H.T., Hanslmayr, S., 2015. Brain oscillations mediate successful suppression of unwanted memories. Cereb Cortex. 25(11), 4180-4190.

Woods, D.L., Kishiyamaa, M.M., Lund, E.W., Herron, T.J., Edwards, B., Poliva, O. et al., 2011. Improving digit span assessment of short-term verbal memory. J Clin Exp Neuropsychol. 33, 101-111.
